# Improved Sleep in Military Personnel is Associated with Changes in the Expression of Inflammatory Genes and Improvement in Depression Symptoms

**DOI:** 10.3389/fpsyt.2015.00059

**Published:** 2015-04-30

**Authors:** Whitney S. Livingston, Heather L. Rusch, Paula V. Nersesian, Tristin Baxter, Vincent Mysliwiec, Jessica M. Gill

**Affiliations:** ^1^National Institutes of Nursing Research, National Institutes of Health, Bethesda, MD, USA; ^2^Johns Hopkins University School of Nursing, Baltimore, MD, USA; ^3^Madigan Army Medical Center, Tacoma, WA, USA

**Keywords:** gene expression, insomnia, inflammation, depression, military personnel

## Abstract

**Study objectives:**

Sleep disturbances are common in military personnel and are associated with increased risk for psychiatric morbidity, including posttraumatic stress disorder (PTSD) and depression, as well as inflammation. Improved sleep quality is linked to reductions in inflammatory bio-markers; however, the underlying mechanisms remain elusive.

**Methods:**

In this study, we examine whole genome expression changes related to improved sleep in 68 military personnel diagnosed with insomnia. Subjects were classified into the following groups and then compared: improved sleep (*n* = 46), or non-improved sleep (*n* = 22) following three months of standard of care treatment for insomnia. Within subject differential expression was determined from microarray data using the Partek Genomics Suite analysis program and the ingenuity pathway analysis (IPA) was used to determine key regulators of observed expression changes. Changes in symptoms of depression and PTSD were also compared.

**Results:**

At baseline, both groups were similar in demographics, clinical characteristics, and gene-expression profiles. The microarray data revealed that 217 coding genes were differentially expressed at the follow-up-period compared to baseline in the participants with improved sleep. Expression of inflammatory cytokines were reduced including IL-1β, IL-6, IL-8, and IL-13, with fold changes ranging from −3.19 to −2.1, and there were increases in the expression of inflammatory regulatory genes including toll-like receptors 1, 4, 7, and 8 in the improved sleep group. IPA revealed six gene networks, including ubiquitin, which was a major regulator in these gene-expression changes. The improved sleep group also had a significant reduction in the severity of depressive symptoms.

**Conclusion:**

Interventions that restore sleep likely reduce the expression of inflammatory genes, which relate to ubiquitin genes and relate to reductions in depressive symptoms.

## Introduction

Military personnel are often exposed to stressful conditions and erratic sleep schedules during deployment, thereby increasing their risk of developing a sleep disorder, most notably insomnia (1, 2). Deployment also increases the risk for psychiatric morbidity, including symptoms of posttraumatic stress disorder (PTSD) and depression, which are often comorbid with insomnia (1, 3–5). Insomnia is the most common symptom for referral in military personnel returning from deployment in Operation Enduring Freedom and Operation Iraqi Freedom (3). Not only is insomnia common but it also results in substantial costs to the individual and society. In the United States, total costs in productivity loss for chronic insomnia (e.g., employment disruption, insomnia-related accidents) are estimated to be between $77 and $92 million each year (6) and total annual costs are estimated to be beyond $100 billion (7). In addition, more than half of military personnel with sleep disturbances present with at least one other medical comorbidity, and those with insomnia are two times more likely to have PTSD (8). Therefore, there is a complex interplay between sleep quality and maintenance of physical and psychiatric health in military personnel, which likely has underlying biological foundations.

Recent studies have begun to determine the relationship of sleep to psychiatric morbidity in those who are under extreme stress (9). For example, pre-deployment sleep disturbance is linked to the onset of depression and PTSD following deployment (10). PTSD includes the symptom of nightmares, which is highly related to insomnia onset and non-remittance (11). Depression also includes symptoms of insomnia including difficulty falling or staying asleep, and these sleep disturbances often do not remit with standard of care treatment for depression (12). Therefore, current studies suggest that there is a shared vulnerability among PTSD, depression, and insomnia, and that these symptoms are often highly related in military personnel who deploy. Seeking care for sleep is often less stigmatizing than mental health care, resulting in military personnel seeking care for insomnia at almost five times the rate as mental health care (13, 14). Therefore, a better understanding of the underlying shared vulnerability of PTSD, depression and insomnia symptoms following deployment may inform interventions to address these comorbidities.

Sleep disturbance, PTSD, and depression are all associated with higher concentrations of inflammatory bio-markers, as well as a greater risk for inflammatory-related morbidities including: metabolic syndrome, cardiovascular disease, obesity, and type II diabetes (15–17). Even short-term reductions in sleep duration in healthy participants increases inflammatory cytokine production, including interleukin-6 (IL-6) (18–20). Chronic insomnia (21), as well as PTSD and depression are associated with increased concentrations of inflammatory cytokines (22). We previously reported that PTSD, depression, and insomnia in military personnel are associated with higher concentrations of inflammatory proteins including C-reactive protein (CRP) (23), and that improved sleep resulted in reductions in inflammation (24). Although these findings support the relationship between sleep and inflammation, the underlying molecular mechanisms of changes in inflammation following sleep improvement remain elusive (2). Gene-expression changes lead to differential protein activity in general; however, there are many other mechanisms that can contribute to the regulation of protein production, and availability, including epigenetic modifications. Therefore, gene-expression changes are one of the first investigations that can explain molecular mechanisms related to proteomic activity changes, and provide novel insights into complex inter-relationships among gene-regulatory mechanisms.

Examination of gene-expression profiles provides insight into how altered gene-activity relates to stress exposure and symptoms. Expression of a gene can increase or decrease and is the initial step to producing proteins that communicate cell function changes, including those active in the immune response system. Combat-related stressors, such as irregular work schedules, traumatic brain injuries, and other life threatening events, are linked to greater concentrations of inflammatory proteins, which are likely a result of gene-expression changes (25). Peripheral blood gene expression is comparable to post-mortem expression in more than 60% of genes, providing a minimally invasive method to investigate gene expression changes in living individuals (26, 27). Peripheral blood has been used to determine that reductions in the expression of circadian rhythm genes, including Clock, brain and muscle Arnt-like protein 1, and Period1, are implicated in symptoms of depression (28). PTSD is also linked to altered gene expression, including reductions in the expression of immune-regulating genes (29); however, the role of sleep and depression were not determined. Identification of inflammatory gene-expression changes related to sleep disturbance might allow for an understanding of the impact of insomnia on other systems and may inform development of targeted interventions to help prevent the development of comorbid disorders and inflammation.

To investigate this critical issue, we used an observational study of military personnel following deployment to determine global gene-expression changes related to improved sleep following 3 months of standard of care treatment. These findings may provide the insight needed to understand relationships between sleep, gene regulation of inflammation, and mental health.

## Materials and Methods

### Study design

This investigation is part of a large ongoing study of U.S. military personnel attending for an initial evaluation of sleep disturbance at the Madigan Army Medical Center Sleep Medicine Clinic in Tacoma, WA, USA. The institutional review board at Madigan Army Medical Center approved the study, and informed consent was obtained from each participant (5). Sixty-eight participants met the study’s inclusion criteria for a sleep diagnosis and completed both pre- and post-treatment assessments; they are included in this analysis. Study participants who met the inclusion criteria were active-duty military personnel who deployed within the previous 18 months with a current diagnosis of insomnia or comorbid insomnia with obstructive sleep apnea (OSA), as previously described (5). Participants were excluded from the study if they had a recent history of drug or alcohol abuse or current diagnosis of bipolar disorder, schizophrenia, or other psychotic disorder. Symptoms related to sleep quality, depression, and PTSD were evaluated through validated instruments at pre-treatment and post-treatment. Participants were classified into one of two groups (improved sleep or non-improved sleep) based on changes in sleep quality from pre- to post-treatment. Both groups were compared on gene-expression changes in inflammatory genes, as well as changes in symptoms of PTSD and depression.

### Assessment and treatment of insomnia and OSA

All participants underwent a clinical evaluation and overnight polysomnography as part of a sleep medicine evaluation using standardized techniques previously reported (5). Depending on their sleep disorder diagnosis, patients were then assigned to a standard of care treatment group. Those with an insomnia diagnosis received 3 months of sleep-focused cognitive behavioral treatment for insomnia (CBT-i), which included aspects of cognitive therapy, stimulus control, sleep restriction, sleep practice (hygiene) education, and time monitoring behavior. Participants diagnosed with comorbid insomnia and OSA received either sleep education or 3 months of CBT-i for their insomnia, and autotitrating positive airway pressure therapy (APAP) for their OSA. A psychologist certified in behavioral sleep medicine or a sleep medicine physician with expertise in CBT-I administered and oversaw all treatments.

### Defining improved sleep

The Pittsburgh sleep quality index (PSQI) was administered to assess participants’ sleep quality and sleep dysfunction over the previous month. This self-report questionnaire has strong diagnostic sensitivity in differentiating good and poor sleepers. Based on a scale of 0 (healthy sleep quality) to 21 (unhealthy sleep quality), a cut-off score of more than 5 is consistent with an insomnia diagnosis (30) and is both reliable (Cronbach’s α = 0.83) and valid (κ = 0.75, *p* < 0.001). For analysis purposes, participants with a reduction in their PSQI score from pre- to post-treatment were assigned to the improved sleep group (*n* = 46). Meanwhile, participants with an increase or no change in PSQI score from pre- to post-treatment were assigned to the non-improved sleep group (*n* = 22).

### Assessment of PTSD and depression

Symptoms of depression were measured using the quick inventory of depressive symptomatology (QIDS). This 16-item inventory has a total possible score range from 0 (lowest severity) to 27 (highest severity). A score of 11, which indicates a moderate severity of depression, was used as the diagnosis cut-off (31). The PTSD checklist-military version (PCL-M) was used to determine symptoms of PTSD (32). A total possible score ranges from 17 (lowest severity) to 85 (highest severity). A total score of 50 and above resulted in a positive PTSD diagnostic screen. The PCL is both reliable and valid compared to the gold standard of the Structured Clinical Interview for DSM-III-R (33).

### mRNA acquisition, quantitation, and hybridization

Blood samples were collected in PAXgene blood RNA tubes and processed with PAXgene™ Blood RNA Kits (PreAnalytiX, Qiagen) for RNA extraction. Quality and quantity of extracted RNA were evaluated with the NanoDrop DN-1000 spectrophotometer (Thermo Fisher Scientific, Wilmington, DE, USA) and the Agilent Bioanalyzer 2100 eukaryotic total RNA Nano assay (Agilent Technologies, Inc., Santa Clara, CA, USA). The 260/280 ratio ranged from 2.03 to 2.34 and the RNA integrity numbers (RIN) were >7.0 in all samples. According to standards (34) a RIN of 6.0 or greater is indicative of admissible quality, and no samples were excluded based on this criterion. Using the GeneChip (GC) 3′ IVT Plus Expression kit, each RNA (100 ng) sample was reverse transcribed, converted to biotinylated cRNA, and hybridized to Affymetrix HG-U133 Plus 2.0 microarrays (Affymetrix, Santa Clara, CA, USA), which contain 54,675 probe-sets representing more than 38,500 specific genes. After staining with streptavidin-phycoerythrin and thorough washing, the raw data were obtained by laser scanning imaging. All assays were undertaken based on standard Affymetrix protocols.

### Statistical analysis

Partek Genomics Suite software, version 6.6 (Partek Inc., St. Louis, MO, USA), was used for all analytic procedures performed on microarray data. Interrogating probes were imported, and corrections for background signal were applied using the robust multi-array average (RMA) method, with additional corrections applied for the GC-content of probes. The probe-sets were standardized using quantile normalization, and expression levels of each probe underwent log-2 transformation to yield distributions of data that more closely approximated normality.

Parameters for identifying differentially expressed genes over time were then identified using analysis of variance (ANOVA) of each probe set’s expression level as a function of time: pre-treatment compared to post-treatment while adjusting for batch effect. A repeated measures comparison was made in each group. Restricted maximum likelihood (REML) method was employed to fit the fixed and random effects of the design separately. Significant differentially expressed gene lists were generated based on criteria of 1.5-fold (up or down) change magnitude and a *p*-value corrected for multiple comparisons using a false discovery rate (FDR) <0.05, for each group.

Two-tailed chi-square tests and independent *t*-tests were used to investigate any pre-treatment group differences that might affect the main analysis. Fisher’s exact tests were used when expected cell counts were <5. The main analysis investigated the relationship between sleep quality improvements or declines and symptoms of PTSD and depression, and gene expression changes following 3 months of sleep treatment. Separate paired *t*-tests were used to examine changes in outcome variables from pre- to post-treatment for both groups. Independent *t*-tests were used to examine between-group differences on outcome variables. The confidence level was set at *p* = 0.05 for all analyses.

QIAGEN’s Ingenuity Pathway Analysis (IPA, QIAGEN Redwood City, www.qiagen.com/ingenuity) was used to determine which diseases and disorders related to observed gene-expression changes, as well as top-gene networks implicated in these gene-expression changes.

## Results

### Demographics and clinical characteristics

The demographic, military, and clinical characteristics of the 68 participants are described in Table 1. As a whole, the sample was primarily male (97%) and Caucasian (62%) with a mean age of 33.8 years (7.8 SD) and 13.8 years (1.8 SD) of education. At pre-treatment, 47% of participants scored positive for depression and 28% scored positive for PTSD. Current use of relevant medications was reported by 40% of the participants. They include antidepressants (28%), pain medications (18%), benzodiazepines (6%), non-benzodiazepines (6%), and the alpha-blocker, prazosin (4%). At pre-treatment, all participants reported a score of at least six on the PSQI, which is a valid indication of poor sleep. There were no between-group differences in demographic variables [i.e., age, gender, race, education, body mass index (BMI), medication use, and military characteristics] with the exception of military rank, for which higher level ranks were more prevalent in the improved sleep group (*p* = 0.015). The prevalence of insomnia, OSA, traumatic brain injury (TBI), depression, and PTSD diagnoses were comparable between groups at baseline and both groups were characterized with high rates of these comorbid symptoms. There was no difference in treatment assignment, either CBT or APAP, between the improved sleep and non-improved sleep groups. The mean score in the PSQI was significantly different, with the improved sleep group having a mean score change of −4.8, whereas the non-improved group had an increase of 2.8 on the PSQI, *p* < 0.01 (see Table 2).

**Table 1:** **Demographic, military, and clinical characteristics**.

	Improved sleep (*n* = 46)	Non-improved sleep (*n* = 22)	*x*^2^*/t*	*p*
Age: mean (SD)	33.5 (7.5)	34.3 (8.7)	0.358	0.722
Males: *n* (%)	45 (97.8)	21 (95.5)	0.293	0.546
Race: *n* (%)			3.663	0.056
Caucasian	32 (69.6)	10 (45.5)		
All others	14 (30.4)	12 (54.5)		
Education in years: mean (SD)	14.0 (2.1)	13.4 (1.3)	−1.202	0.234
Rank: *n* (%)			5.921	0.015
Lower	26 (56.5)	19 (86.4)		
Senior or Officer	20 (43.5)	3 (13.6)		
Front line military exposure: *n* (%)	17 (37.0)	9 (40.9)	0.098	0.754
Number of deployments: *n* (%)			0.718	0.397
1 or 2	30 (65.2)	12 (54.5)		
3 or more	16 (34.8)	10 (45.5)		
Time since deployment: *n* (%)			0.729	0.393
<6 months	14 (30.4)	9 (40.9)		
>6 months	32 (69.6)	13 (59.1)		
BMI: mean (SD)	30.2 (4.3)	30.5 (3.4)	0.330	0.743
Medication use: *n* (%)	16 (34.8)	11 (50.0)	1.440	0.230
OSA diagnosis: *n* (%)	12 (26.1)	5 (22.7)	0.090	0.765
TBI diagnosis: *n* (%)	18 (39.1)	9 (40.9)	0.020	0.888
Probable depression diagnosis: *n* (%)	21 (45.7)	11 (50.0)	0.113	0.737
Probable PTSD diagnosis: *n* (%)	11 (23.9)	8 (36.4)	1.146	0.284

*BMI, body mass index; OSA, obstructive sleep apnea; PTSD, posttraumatic stress disorder; TBI, traumatic brain injury*.

*Fisher’s exact test was used when expected cell counts were <5*.

**Table 2:** **Pre- and post-treatment symptom severity mean (SD)**.

	Improved sleep (*n* = 46)			Non-improved sleep (*n* = 22)			Between-group	
	Pre-treatment	Post-treatment	*p*	Pre-treatment	Post-treatment	*p*	Baseline	Change^a^
	
							*p*	*p*
Sleep quality	12.8 (4.1)	8.0 (3.9)	**<0.001**	12.1 (3.6)	14.9 (3.1)	**<0.001**	0.484	**<0.001**
Depression severity	10.5 (5.3)	8.1 (5.0)	**0.001**	11.00 (4.7)	12.2 (5.0)	0.134	0.732	**0.002**
PTSD severity	38.7 (15.3)	35.6 (16.5)	0.067	43.5 (16.2)	46.5 (17.6)	0.223	0.237	**0.040**

*^a^Pre- to post-treatment change-score. Bold font indicates findings with significant change*.

### Psychological symptom change

Sleep quality, depression, and PTSD outcomes were assessed before and after 3 months of sleep treatment. Table 2 details the pre- and post-treatment symptom severity of both groups. Briefly, from pre- to post-treatment, the improved sleep group reported significant reductions in depression [*t*(45) = 3.463, *p* = 0.001] and a trend for decreases in posttraumatic symptoms [*t*(45) = 1.875, *p* = 0.067]. These improvements were significant between groups (all *p*-values <0.05). Meanwhile, the non-improved sleep group reported no significant change in depression (*p* = 0.134) or posttraumatic symptoms (*p* = 0.223).

### Gene-expression profiles

The microarray data revealed that 217 coding genes (Table 3) were differentially expressed at the follow-up-period compared to baseline in the participants who improved in sleep. Of these 217 genes, 113 genes were down-regulated (Table 4) and 104 were up-regulated (Table 5). None of these genes changed significantly in the non-improved sleep group.

**Table 3:** **All differentially expressed genes**.

Gene symbol	Gene title	Fold-change	*p*-Value
CCL4	Chemokine (C—C motif) ligand 4	−3.38991	0.000400502
EGR3	Early growth response 3	−3.33259	4.37E-05
IL1B	Interleukin 1, beta	−3.19348	0.00054254
TIGIT	T cell immunoreceptor with Ig and ITIM domains	−2.999421	0.000116146
CD96	CD96 molecule	−2.9992104	0.000593266
CCL3///CCL3L1///CCL3L3	Chemokine (C—C motif) ligand 3///chemokine (C—C motif) ligand 3-like 1///chemokine	−2.99786	1.32E-05
CCL5	Chemokine (C—C motif) ligand 5	−2.96621	0.000882838
TNF	Tumor necrosis factor	−2.90578	0.000536776
DUSP1	Dual specificity phosphatase 1	−2.89657	1.73E-05
GATA3	GATA binding protein 3	−2.889336	0.000852941
PTGS2	Prostaglandin-endoperoxide synthase 2 (prostaglandin G/H synthase and cyclooxygenase)	−2.86159	0.000116328
BZRAP1	Benzodiazepine receptor (peripheral) associated protein 1	−2.84583	0.000429033
KIR3DL1///KIR3DL2///LOC727787	Killer cell immunoglobulin-like receptor, three domains, long cytoplasmic tail, 1///k	−2.79571	0.00111701
IL18R1	Interleukin 18 receptor 1	−2.79239	0.00131446
NFATC2	Nuclear factor of activated T-cells, cytoplasmic, calcineurin-dependent 2	−2.77617	0.000468791
IFITM1	Interferon Interferon-induced transmembrane protein 1 (9—27)	−2.67642	0.000796847
IL6	Interleukin 6 (interferon, beta 2)	−2.66587	0.000169597
IFITM1	Interferon Interferon-induced transmembrane protein 1 (9—27)	−2.59115	0.00120357
GLCCI1	Glucocorticoid Glucocorticoid-induced transcript 1	−2.57928	0.000863656
PER1	Period homolog 1 (*Drosophila*)	−2.34547	0.000172781
TARP///TRGC2	TCR gamma alternate reading frame protein///T cell receptor gamma constant 2	−2.31319	0.000858636
TARP	TCR gamma alternate reading frame protein	−2.2425	0.00123683
TP53INP2	Tumor protein p53 inducible nuclear protein 2	−2.23152	2.18E-04
CDC14A	CDC14 cell division cycle 14 homolog A (*S. cerevisiae*)	−2.22332	0.00055414
ZNF831	Zinc finger protein 831	−2.21191	0.000940436
BCL10	B-cell CLL/lymphoma 10	−2.19636	1.92E-04
TARP///TRGC2	TCR gamma alternate reading frame protein///T cell receptor gamma constant 2	−2.18635	0.00138229
TARP///TRGC2	TCR gamma alternate reading frame protein///T cell receptor gamma constant 2	−2.17218	0.00136634
STAT3	Signal transducer and activator of transcription 3 (acute-phase response factor)	−2.17143	2.79E-05
IL13	Interleukin 13	−2.0998	0.000453301
ZNF831	Zinc finger protein 831	−2.07393	0.000998177
GZMB	Granzyme B (granzyme 2, cytotoxic T-lymphocyte-associated serine esterase 1)	−2.07291	0.000106599
KLF12	Kruppel-like factor 12	−2.06742	0.000474026
SPON2	Spondin 2, extracellular matrix protein	−2.06256	0.000562071
MBNL2	Muscleblind-like 2 (*Drosophila*)	−2.06215	0.000146908
KRAS	v-Ki-ras2 Kirsten rat sarcoma viral oncogene homolog	−2.04976	0.000826814
CRA	Complement factor C4	−2.04778987	7.09143E-05
FCAR	Fc fragment of IgA, receptor for	−2.03608	1.89E-04
NCL	Nucleolin	−2.0312	4.39101E-07
IFRD1	Interferon-related developmental regulator 1	−2.02534	7.16E-06
S1PR5	Sphingosine-1-phosphate receptor 5	−1.99987	0.000877998
NAP1L5	Nucleosome assembly protein 1-like 5	−1.9825	0.00103436
FGFBP2	Fibroblast growth factor binding protein 2	−1.95856	0.00097833
TRD@	T cell receptor delta locus	−1.95283	0.00111344
PYHIN1	Pyrin and HIN domain family, member 1	−1.93462	0.000986752
GFI1	Growth factor independent 1 transcription repressor	−1.93194	0.00076425
GZMM	Granzyme M (lymphocyte met-ase 1)	−1.93125	0.00085383
TRD@	T cell receptor delta locus	−1.9207	0.00111727
TSC22D3	TSC22 domain family, member 3	−1.91096	2.28E-06
KLRB1	Killer cell lectin-like receptor subfamily B, member 1	−1.90964	0.00136588
IL18RAP	Interleukin 18 receptor accessory protein	−1.90962	0.000221487
ICOS	Inducible co-stimulator	−1.903002	0.000880293
MYLIP	Myosin regulatory light chain interacting protein	−1.89332	8.32E-06
TGFBR3	Transforming growth factor, beta receptor III	−1.8931	0.00121075
ZAP70	Zeta-chain (TCR) associated protein kinase 70 kDa	−1.89061	0.000662326
CTSW	Cathepsin W	−1.88397	0.00129928
CEP78	Centrosomal protein 78 kDa	−1.88364	0.000127057
ARL4C	ADP-ribosylation factor-like 4C	−1.8832	0.000160201
ARL4C	ADP-ribosylation factor-like 4C	−1.87226	0.000419378
KLRD1	Killer cell lectin-like receptor subfamily D, member 1	−1.86593	0.00124982
PRKCQ	Protein kinase C, theta	−1.86552	0.00113571
NR4A1	Nuclear receptor subfamily 4, group A, member 1	−1.86138	8.90E-05
SYTL2	Synaptotagmin-like 2	−1.85889	0.00135142
ARL4C	ADP-ribosylation factor-like 4C	−1.85424	0.000286602
AGAP1	ArfGAP with GTPase domain, ankyrin repeat and PH domain 1	−1.80837	0.000287414
SH2D2A	SH2 domain containing 2A	−1.80828	0.000300214
KLRK1	Killer cell lectin-like receptor subfamily K, member 1	−1.78448	0.00102739
RUNX3	Runt-related transcription factor 3	−1.77892	0.00013984
KIR3DL3	Killer cell immunoglobulin-like receptor, three domains, long cytoplasmic tail, 3	−1.70915	0.000885828
PER1	Period homolog 1 (*Drosophila*)	−1.70146	0.000444082
CHD2	Chromodomain helicase DNA DNA-binding protein 2	−1.70091	0.000257214
DTX3	Deltex homolog 3 (*Drosophila*)	−1.70068	0.000829046
PRKCH	Protein kinase C, eta	−1.68436	0.00106358
FAM179A	Family with sequence similarity 179, member A	−1.68265	0.000135121
MGAT4A	Mannosyl (alpha-1,3-)-glycoprotein beta-1,4-*N*-acetylglucosaminyltransferase, isozyme A	−1.67906	0.000326335
GBP5	Guanylate binding protein 5	−1.67871	0.000452814
MLC1	Megalencephalic leukoencephalopathy with subcortical cysts 1	−1.66993	0.000433367
ANKRD36	Ankyrin repeat domain 36	−1.63932	0.000866547
NFATC2	Nuclear factor of activated T-cells, cytoplasmic, calcineurin-dependent 2	−1.63898	0.000941657
SYNE2	Spectrin repeat containing, nuclear envelope 2	−1.63663	1.19E-05
ID2	Inhibitor of DNA DNA-binding 2, dominant negative helix—loop—helix protein	−1.60803	1.67E-06
KIF21A	Kinesin family member 21A	−1.60799	0.00136965
CTNNB1	Catenin (cadherin-associated protein), beta 1, 88 kDa	−1.6076	4.09E-05
TSEN54	tRNA splicing endonuclease 54 homolog (*S. cerevisiae*)	−1.60547	0.000604728
ZNF451	Zinc finger protein 451	−1.60463	6.28E-06
TSPAN5	Tetraspanin 5	−1.60208	0.00114212
RASGEF1A	RasGEF domain family, member 1A	−1.60149	0.000447691
TNIK	TRAF2 and NCK interacting kinase	−1.59894	2.50E-05
CBLB	Cas-Br-M (murine) ecotropic retroviral transforming sequence b	−1.5979	0.000320734
MLLT11	Myeloid/lymphoid or mixed-lineage leukemia (trithorax homolog, *Drosophila*); translocate	−1.59317	0.000831119
TAF9B	TAF9B RNA polymerase II, TATA box binding protein (TBP)-associated factor, 31 kDa	−1.5885	0.000321819
HNRNPL	Heterogeneous nuclear ribonucleoprotein L	−1.5871	3.77E-06
SESN2	Sestrin 2	−1.57849	6.97E-05
LOC100130872	Hypothetical LOC100130872	−1.56568	1.48E-05
SESN2	Sestrin 2	−1.56417	0.00050745
CCL5	Chemokine (C—C motif) ligand 5	−1.56409	0.000430274
PIK3R1	Phosphoinositide-3-kinase, regulatory subunit 1 (alpha)	−1.56311	0.00143535
TFDP2	Transcription factor Dp-2 (E2F dimerization partner 2)	−1.55293	0.000804861
PDE4B	Phosphodiesterase 4B, cAMP-specific	−1.55035	0.00085501
RBM14	RNA binding motif protein 14	−1.54638	2.35E-05
FAM100B	Family with sequence similarity 100, member B	−1.53662	7.63E-05
PDE4D	Phosphodiesterase 4D, cAMP-specific	−1.53463	0.000469774
GRPEL1	GrpE-like 1, mitochondrial (*E. coli*)	−1.53133	1.23E-05
ZNF507	Zinc finger protein 507	−1.52977	0.000202111
NUP153	Nucleoporin 153 kDa	−1.52758	0.000367042
FUS	Fused in sarcoma	−1.52544	0.0010451
PTTG1	Pituitary tumor-transforming 1	−1.52511	0.000818797
WAC	WW domain containing adaptor with coiled-coil	−1.52328	0.000534293
EIF1	Eukaryotic translation initiation factor 1	−1.52229	6.21E-06
TSEN54	tRNA splicing endonuclease 54 homolog (*S. cerevisiae*)	−1.50904	0.000620767
MYLIP	Myosin regulatory light chain interacting protein	−1.50894	3.24E-05
CALU	Calumenin	−1.50768	0.000736822
EHD4	EH-domain containing 4	−1.50216	0.000209267
ATP11B	ATPase, class VI, type 11B	1.50127	0.000202173
FKBP15	FK506 binding protein 15, 133 kDa	1.50442	0.000270622
MOCS3	Molybdenum cofactor synthesis 3	1.5075	4.38E-06
FAM13A	Family with sequence similarity 13, member A	1.50801	0.00135619
CDC42EP3	CDC42 effector protein (Rho GTPase binding) 3	1.50849	2.59E-05
DACH1	Dachshund homolog 1 (*Drosophila*)	1.51638	0.000969487
HHEX	Hematopoietically expressed homeobox	1.51675	6.21E-05
PDE7B	Phosphodiesterase 7B	1.51869	2.37E-05
OBFC2A	Oligonucleotide/oligosaccharide-binding fold containing 2A	1.52002	0.000343088
CR1	Complement component (3b/4b) receptor 1 (Knops blood group)	1.52311	9.05E-05
PHF23	PHD finger protein 23	1.53301	3.41E-06
ZNF780A	Zinc finger protein 780A	1.53325	0.000505801
GPR27	G protein-coupled receptor 27	1.53487	0.0011538
CDC42EP3	CDC42 effector protein (Rho GTPase binding) 3	1.53614	1.72E-05
ADPRH	ADP-ribosylarginine hydrolase	1.53673	0.000301124
TLR4	Toll-like receptor 4	1.54384	0.000155809
TLR10	Toll-like receptor 10	1.54391	0.000150659
SIRPB2	Signal-regulatory protein beta 2	1.55351	9.39E-05
FUBP1	Far upstream element (FUSE) binding protein 1	1.55437	3.43E-05
NLRC4	NLR family, CARD domain containing 4	1.5579	0.0010619
OBFC2A	Oligonucleotide/oligosaccharide-binding fold containing 2A	1.55822	6.88E-05
TRPS1	Trichorhinophalangeal syndrome I	1.56073	0.00117806
CLIC4	Chloride intracellular channel 4	1.56137	0.00142631
ANKRD57	Ankyrin repeat domain 57	1.56891	0.000496592
FOSL2	FOS-like antigen 2	1.57091	0.00103799
FBXO30	F-box protein 30	1.57898	3.37E-05
S1PR3	Sphingosine-1-phosphate receptor 3	1.57953	0.000766403
MFAP3	Microfibrillar-associated protein 3	1.58049	8.30E-05
CPD	Carboxypeptidase D	1.58561	0.000270503
RNF24	Ring finger protein 24	1.5887	0.000779404
PIGM	Phosphatidylinositol glycan anchor biosynthesis, class M	1.58922	3.93E-06
SAP30L	SAP30-like	1.599	3.48E-05
SERPINI2	Serpin peptidase inhibitor, clade I (pancpin), member 2	1.60236	0.000725023
BACH1	BTB and CNC homology 1, basic leucine zipper transcription factor 1	1.60519	5.89E-06
ZNF322B	Zinc finger protein 322B	1.60589	0.000435523
ZEB2	Zinc finger E-box binding homeobox 2	1.61326	0.0010863
GPATCH2	G patch domain containing 2	1.61607	0.000829918
KCNE3	Potassium voltage-gated channel, Isk-related family, member 3	1.62682	0.000871206
ENTPD1	Ectonucleoside triphosphate diphosphohydrolase 1	1.62899	0.000688504
FAM114A1	Family with sequence similarity 114, member A1	1.63805	0.000957221
ZNF697	Zinc finger protein 697	1.63815	0.000362559
SIPA1L1	Signal-induced proliferation-associated 1 1-like 1	1.63837	0.000200843
MGST1	Microsomal glutathione S-transferase 1	1.63932	0.000155808
CENPBD1	CENPB DNA-binding domains containing 1	1.63975	0.000598446
LOC100505956	Hypothetical LOC100505956	1.6413	0.00103297
CTTNBP2NL	CTTNBP2 N-terminal like	1.6428	0.00126079
CLLU1	Chronic lymphocytic leukemia up-regulated 1	1.65206	0.000787124
ST8SIA4	ST8 alpha-*N*-acetyl-neuraminide alpha-2,8-sialyltransferase 4	1.66224	0.000130372
FAR2	Fatty acyl CoA reductase 2	1.68735	5.72E-05
DAPP1	Dual adaptor of phosphotyrosine and 3-phosphoinositides	1.68966	0.000609128
PLD1	Phospholipase D1, phosphatidylcholine-specific	1.69193	0.000517971
MGST1	Microsomal glutathione S-transferase 1	1.69246	0.00020858
MGST1	Microsomal glutathione S-transferase 1	1.69458	7.19E-06
DSC2	Desmocollin 2	1.69681	0.000254904
GPATCH2	G patch domain containing 2	1.70199	0.000112569
ST6GALNAC3	ST6 (alpha-*N*-acetyl-neuraminyl-2,3-beta-galactosyl-1,3)-*N*-acetylgalactosaminide alpha-2	1.70328	0.000428429
FBN2	Fibrillin 2	1.721	0.000252441
FCGR1B	Fc fragment of IgG, high affinity Ib, receptor (CD64)	1.72442	0.000706171
HP	Haptoglobin	1.72582	0.000718758
FRAT2	Frequently rearranged in advanced T-cell lymphomas 2	1.72608	0.000190515
HHEX	Hematopoietically expressed homeobox	1.7329	4.10E-06
P2RY13	Purinergic receptor P2Y, G-protein coupled, 13	1.73405	0.000133887
MGST1	Microsomal glutathione S-transferase 1	1.74784	7.56E-05
LOC100506828	Hypothetical LOC100506828	1.75228	7.44E-05
TFEC	Transcription factor EC	1.75494	0.000135064
WLS	Wntless homolog (*Drosophila*)	1.76165	0.000227731
FCGR1A///FCGR1C	Fc fragment of IgG, high affinity Ia, receptor (CD64)///Fc fragment of IgG, high affinity	1.76282	0.00095328
SGMS2	Sphingomyelin synthase 2	1.76728	0.000618004
ATP6V1A	ATPase, H + transporting, lysosomal 70 kDa, V1 subunit A	1.77021	3.21E-05
FPR2	Formyl peptide receptor 2	1.77041	8.98E-05
ADAM9	ADAM metallopeptidase domain 9	1.82176	2.72E-05
TMEM49	Transmembrane protein 49	1.8221	0.000516735
WSB1	WD repeat and SOCS box-containing 1	1.82444	0.000178707
NR3C1	Promoter 1B of the GR	1.83029221	0.00019027
ADAMTS5	ADAM metallopeptidase with thrombospondin type 1 motif, 5	1.83321	0.000299013
FPR2	Formyl peptide receptor 2	1.85476	0.000499535
EMP1	Epithelial membrane protein 1	1.8738	0.000128519
SPATA5L1	Spermatogenesis associated 5-like 1	1.87522	0.00102786
PLA2G4A	Phospholipase A2, group IVA (cytosolic, calcium-dependent)	1.87895	0.000196578
TLR1	Toll-like receptor 1	1.93858	2.14E-07
LOC203274	Hypothetical protein LOC203274	1.94801	5.09E-05
NFATC2	Nuclear factor of activated T-cells, cytoplasmic, calcineurin-dependent 2	1.9776	5.93E-05
FBXO30	F-box protein 30	1.98992	0.000899931
ATP6V1A	ATPase, H + transporting, lysosomal 70 kDa, V1 subunit A	1.99	0.000810192
CYP1B1	Cytochrome P450, family 1, subfamily B, polypeptide 1	1.99311	9.37E-06
STEAP4	STEAP family member 4	2.01298	0.000150426
IL8R1	Interleukin 8 receptor 1	2.03E + 00	3.92893E-05
IL2	interleukin 2	2.03419987	3.10905E-06
FOLR3	Folate receptor 3 (gamma)	2.05781	0.00017189
FKBP15	FK506 binding protein 15, 133 kDa	2.094500914	4.30009E-05
SLITRK4	SLIT and NTRK-like family, member 4	2.16832	6.47E-05
ADAMTS5	ADAM metallopeptidase with thrombospondin type 1 motif, 5	2.18224	0.00016302
PTPRO	Protein tyrosine phosphatase, receptor type, O	2.21369	0.000260765
FAM198B	Family with sequence similarity 198, member B	2.22299	0.000661349
FKBP506	FKBP506 binding protein	2.223221099	1.90294E-06
IFNGR1	Interferon gamma receptor 1	2.51823	0.000195788
TLR7	Toll-like receptor 7	2.59205	0.00115974
RIN2	Ras and Rab interactor 2	2.62017	0.000313438
TLR8	Toll-like receptor 8	2.62471	0.00112645
TLR4	Toll-like receptor 4	2.66149	0.000182505
MIR21	MicroRNA 21	2.68409	0.000265287
CYP1B1	Cytochrome P450, family 1, subfamily B, polypeptide 1	2.83233	0.000146054
PDK4	Pyruvate dehydrogenase kinase, isozyme 4	2.99062	7.43E-07
PDK4	Pyruvate dehydrogenase kinase, isozyme 4	3.1238	0.000118651

**Table 4:** **Down-regulated gene expression (top 50)**.

Gene symbol	Gene title	Fold-change	*p*-Value
CCL4	Chemokine (C–C motif) ligand 4	−3.39	0.0004
EGR3	Early growth response 3	−3.333	4.37E-05
IL1B	Interleukin 1, beta	−3.193	0.00054
TIGIT	T cell immunoreceptor with Ig and ITIM domains	−2.999	0.00012
CD96	CD96 molecule	−2.999	0.00059
CCL3///CCL3L1///CCL3L3	Chemokine (C–C motif) ligand 3///chemokine (C–C motif) ligand 3-like 1///chemokine	−2.998	1.32E-05
CCL5	Chemokine (C–C motif) ligand 5	−2.966	0.00088
TNF	Tumor necrosis factor	−2.906	0.00054
DUSP1	Dual specificity phosphatase 1	−2.897	1.73E-05
GATA3	GATA binding protein 3	−2.889	0.00085
PTGS2	Prostaglandin-endoperoxide synthase 2 (prostaglandin G/H synthase and cyclooxygenase)	−2.862	0.00012
BZRAP1	Benzodiazepine receptor (peripheral) associated protein 1	−2.846	0.00043
KIR3DL1///KIR3DL2///LOC727787	Killer cell immunoglobulin-like receptor, three domains, long cytoplasmic tail, 1///k	−2.796	0.00112
IL18R1	Interleukin 18 receptor 1	−2.792	0.00131
NFATC2	Nuclear factor of activated T-cells, cytoplasmic, calcineurin-dependent 2	−2.776	0.00047
IFITM1	Interferon-induced transmembrane protein 1 (9–27)	−2.676	0.0008
IL6	Interleukin 6 (interferon, beta 2)	−2.666	0.00017
IFITM1	Interferon-induced transmembrane protein 1 (9–27)	−2.591	0.0012
GLCCI1	Glucocorticoid induced transcript 1	−2.579	0.00086
PER1	Period homolog 1 (*Drosophila*)	−2.345	0.00017
TARP///TRGC2	TCR gamma alternate reading frame protein///T cell receptor gamma constant 2	−2.313	0.00086
TARP	TCR gamma alternate reading frame protein	−2.243	0.00123683
TP53INP2	Tumor protein p53 inducible nuclear protein 2	−2.232	2.18E-04
CDC14A	CDC14 cell division cycle 14 homolog A (*S. cerevisiae*)	−2.223	0.00055
ZNF831	Zinc finger protein 831	−2.212	0.00094
BCL10	B-cell CLL/lymphoma 10	−2.196	1.92E-04
TARP///TRGC2	TCR gamma alternate reading frame protein///T cell receptor gamma constant 2	−2.186	0.00138
TARP///TRGC2	TCR gamma alternate reading frame protein///T cell receptor gamma constant 2	−2.172	0.00137
STAT3	Signal transducer and activator of transcription 3 (acute-phase response factor)	−2.171	2.79E-05
IL13	Interleukin 13	−2.1	0.00045
ZNF831	Zinc finger protein 831	−2.074	0.001
GZMB	Granzyme B (granzyme 2, cytotoxic T-lymphocyte-associated serine esterase 1)	−2.073	0.00011
KLF12	Kruppel-like factor 12	−2.067	0.00047
SPON2	Spondin 2, extracellular matrix protein	−2.063	0.00056
MBNL2	Muscleblind-like 2 (*Drosophila*)	−2.062	0.00015
KRAS	v-Ki-ras2 Kirsten rat sarcoma viral oncogene homolog	−2.05	0.00083
CRA	Complement factor C4	−2.048	7.09E-05
FCAR	Fc fragment of IgA, receptor for	−2.036	1.89E-04
NCL	Nucleolin	−2.031	4.39E-07
IFRD1	Interferon-related developmental regulator 1	−2.025	7.16E-06
S1PR5	Sphingosine-1-phosphate receptor 5	−2.00	0.00088
NAP1L5	Nucleosome assembly protein 1-like 5	−1.983	0.00103
FGFBP2	Fibroblast growth factor binding protein 2	−1.959	0.00098
TRD	T cell receptor delta locus	−1.953	0.00111
PYHIN1	Pyrin and HIN domain family, member 1	−1.935	0.00099
GFI1	Growth factor independent 1 transcription repressor	−1.932	0.00076
GZMM	Granzyme M (lymphocyte met-ase 1)	−1.931	0.00085
TRD	T cell receptor delta locus	−1.921	0.00112
TSC22D3	TSC22 domain family, member 3	−1.911	2.28E-06
KLRB1	Killer cell lectin-like receptor subfamily B, member 1	−1.91	0.00137

**Table 5:** **Up-regulated gene expression (top 50)**.

Gene symbol	Gene title	Fold-change	*p*-Value
PDK4	Pyruvate dehydrogenase kinase, isozyme 4	3.1238	0.00012
CYP1B1	Cytochrome P450, family 1, subfamily B, polypeptide 1	2.83233	0.00015
MIR21	MicroRNA 21	2.68409	0.00027
TLR4	Toll-like receptor 4	2.66149	0.00018
TLR8	Toll-like receptor 8	2.62471	0.00113
RIN2	Ras and Rab interactor 2	2.62017	0.00031
TLR7	Toll-like receptor 7	2.59205	0.00116
IFNGR1	Interferon gamma receptor 1	2.51823	0.0002
FKBP506	FKBP506 binding protein	2.22322	1.90E-06
FAM198B	Family with sequence similarity 198, member B	2.22299	0.00066
PTPRO	Protein tyrosine phosphatase, receptor type, O	2.21369	0.00026
ADAMTS5	ADAM metallopeptidase with thrombospondin type 1 motif, 5	2.18224	0.00016
SLITRK4	SLIT and NTRK-like family, member 4	2.16832	6.47E-05
FKBP15	FK506 binding protein 15, 133 kDa	2.0945	4.30E-05
FOLR3	Folate receptor 3 (gamma)	2.05781	0.00017
IL2	Interleukin 2	2.0342	3.10E-06
IL8R1	Interleukin 8 receptor 1	2.03E + 00	3.90E-05
STEAP4	STEAP family member 4	2.01298	0.00015
ATP6V1A	ATPase, H+ transporting, lysosomal 70 kDa, V1 subunit A	1.99	0.00081
FBXO30	F-box protein 30	1.98992	0.0009
NFATC2	Nuclear factor of activated T-cells, cytoplasmic, calcineurin-dependent 2	1.9776	5.90E-05
LOC203274	Hypothetical protein LOC203274	1.94801	5.09E-05
TLR1	Toll-like receptor 1	1.93858	2.14E-07
PLA2G4A	Phospholipase A2, group IVA (cytosolic, calcium-dependent)	1.87895	0.0002
SPATA5L1	Spermatogenesis associated 5-like 1	1.87522	0.00103
EMP1	Epithelial membrane protein 1	1.8738	0.00013
FPR2	Formyl peptide receptor 2	1.85476	0.0005
ADAMTS5	ADAM metallopeptidase with thrombospondin type 1 motif, 5	1.83321	0.0003
NR3C1	Promoter 1B of the GR	1.83029	0.00019
WSB1	WD repeat and SOCS box-containing 1	1.82444	0.00018
TMEM49	Transmembrane protein 49	1.8221	0.00052
ADAM9	ADAM metallopeptidase domain 9	1.82176	2.72E-05
FPR2	Formyl peptide receptor 2	1.77041	8.98E-05
ATP6V1A	ATPase, H+ transporting, lysosomal 70 kDa, V1 subunit A	1.77021	3.21E-05
SGMS2	Sphingomyelin synthase 2	1.76728	0.00062
FCGR1A///FCGR1C	Fc fragment of IgG, high affinity Ia, receptor (CD64)///Fc fragment of IgG, high affinity	1.76282	0.00095
WLS	Wntless homolog (*Drosophila*)	1.76165	0.00023
TFEC	Transcription factor EC	1.75494	0.00014
LOC100506828	Hypothetical LOC100506828	1.75228	7.44E-05
MGST1	Microsomal glutathione S-transferase 1	1.74784	7.56E-05
P2RY13	Purinergic receptor P2Y, G-protein coupled, 13	1.73405	0.00013
HHEX	Hematopoietically expressed homeobox	1.7329	4.10E-06
FRAT2	Frequently rearranged in advanced T-cell lymphomas 2	1.72608	0.00019
HP	Haptoglobin	1.72582	0.00072
FCGR1B	Fc fragment of IgG, high affinity Ib, receptor (CD64)	1.72442	0.00071
FBN2	Fibrillin 2	1.721	0.00025
ST6GALNAC3	ST6 (alpha-*N*-acetyl-neuraminyl-2,3-beta-galactosyl-1,3)-*N*-acetylgalactosaminide alpha-2	1.70328	0.00043
GPATCH2	G patch domain containing 2	1.70199	0.00011
DSC2	Desmocollin 2	1.69681	0.00025
MGST1	Microsomal glutathione S-transferase 1	1.69458	7.19E-06

Expression of inflammatory cytokines were substantially reduced in the improved sleep group, including interleukin 1 beta (IL-1β), interleukin 6 (IL-6), interleukin 8 (IL-8), and interleukin 13 (IL-13), with fold changes ranging from −3.19 to −2.10. We also report significant reductions in chemokine genes including chemokine (C–C motif) ligand 4 (CCL4), which was the most substantially reduced gene, with a fold change of −3.39, as well as chemokine (C–C motif) ligand 3 (CCL3), and chemokine (C–C motif) ligand 5 (CCL5), with fold-changes of −3.00 and −2.97, respectively, in the improved sleep group.

Genes with increases in expression in the improved sleep group were predominately inflammatory regulatory genes including toll-like receptors 4 and 8 (TLR4 and TLR8), with a 2.66- and 2.62-fold change, respectively, as well as toll-like receptors 7 and 1 (TLR7 and TLR1), with fold changes of 2.6 and 1.9, respectively. Stress-related genes of cytochrome P450, family 1, subfamily B, polypeptide 1 (CYP1B1), FKBP506 binding protein (FKBP506), and FK506 binding protein 15, 133 kDa (FKBP15) exhibited fold changes >2.0. The pyruvate dehydrogenase kinase, isozyme 4 genes had the greatest increase in expression, with a fold change of 3.12.

**Figure 1:**
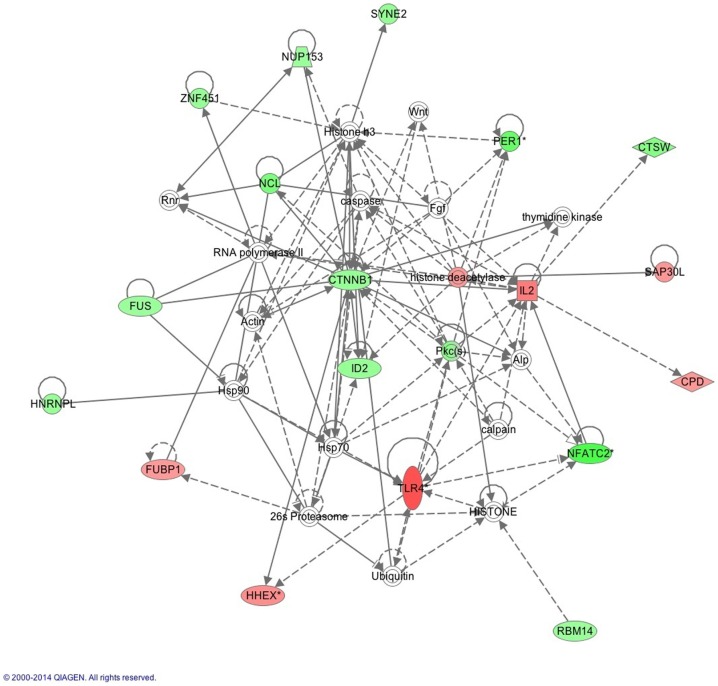
**This network shows genes related to cellular development and connective tissue**. Generated molecular network based on differential expression in the improved sleep group from baseline to follow-up using ingenuity knowledge database. Coloring is based on the expression values of the genes (fold changes shown): down-regulation in green and up-regulation in red. Genes with no coloring are added from ingenuity knowledge database. Direct and indirect relationships are shown by solid and dashed lines, respectively. The arrow indicates specific directionality of interactions. Genes with an asterisk indicate that multiple identifiers (probesets) map to the gene in the molecular network. Reprinted with permission from QIAGEN’s Ingenuity^®^ Pathway Analysis (IPA^®^) (http://www.ingenuity.com/).

**Figure 2:**
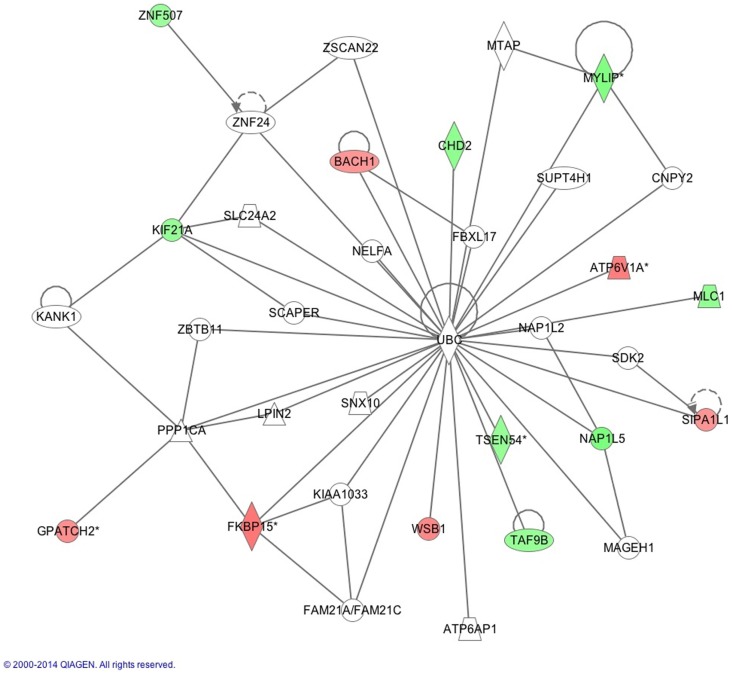
**This network shows genes related to developmental disorders, as reported by IPA**. Reprinted with permission from QIAGEN’s Ingenuity^®^ Pathway Analysis (IPA^®^) (http://www.ingenuity.com/).

The IPA report shows conical pathways were altered and related to improved sleep. The pathways include the inflammatory response, infectious disease, inflammatory disease, and organismal injury and abnormalities. There were six significant gene-networks identified, the scores of which are reported in Table 6. Networks 1 and 2 are provided in Figures 1 and 2, and the remaining Figures S1–S4 are provided in the Supplementary Material. With the exception of the third IPA network, the other five contained genes above a fold change of 2 in the networks reported. Network 1 includes genes related to cellular development and tissue disorders, and has a score of 29. In this network, there are three main hubs: Catenin (cadherin-associated protein), beta 1, 88 kDa (CTNNβ1), toll-like receptors (TLR), and interleukin 2 (IL-2), as well as minor hubs of ubiquitin (UBC), polyketide synthase (PKS), and nuclear factor of activated T-cells 2 (NFATC2). Network 2 has a score of 23, and includes genes related to cancer, and tissue and developmental disorders. This network has a primary hub of UBC, which is related to an increase in expression of FKBP15 and ATPase, H+ transporting, lysosomal 70 kDa, and V1 subunit A (ATP6V1A), and reductions in the expression of TSEN54 tRNA splicing endonuclease and megalencephalic leukoencephalopathy with subcortical cysts 1.

**Table 6:** **IPA network scores**.

Networks	Score
1	29
2	14
3	14
4	14
5	13
6	14

*This table lists the scores of the six networks (Figures 1 and 2; Figures S1–S4 in Supplementary Material) as reported by ingenuity pathway analysis (IPA). Following the compilation of networks, based on the interconnectivity of each gene, IPA finalizes the report by ranking each network based on a calculation of *p*-scores [*p*-score = −log_10_(*p*-value)]. This score accounts for the number of genes versus the size of the network to estimate how valid this network is to the original list of genes. This allows for the networks to be prioritized as seen in Table 6, where a score above 20 is seen as more significant*.

## Discussion

The primary aim of the current study is to examine the relationship between changes in self-reported sleep quality and changes in expression of inflammatory genes and symptoms of depression and PTSD following 3 months of sleep treatment in military personnel with insomnia and comorbid psychiatric disorders. At post-treatment, participants with improved sleep have significantly reduced depression symptoms and changes in gene expression, which most notably reflect reductions in inflammatory cytokine genes. These novel findings provide unique insights into our previous findings, which indicated improved sleep quality is associated with reductions in concentrations of CRP and improvements in health-related quality of life in military personnel (23). These results suggest that sleep-focused treatments effectively reduce inflammatory gene activity, and may attenuate the risk for inflammatory-related morbidity in this population.

Specifically, we report that gene activity is reduced in 113 genes following improved sleep, including significant reductions in the expression of the following inflammatory cytokine genes: CCL4, IL-1β, tumor necrosis factor (TNF), IL-6, and IL-13 (Table 4). Elevated concentrations of IL-6 have been associated with symptoms of insomnia, PTSD, and depression (22, 35). The reduction in IL-6 secretion has significant ramifications as IL-6 has a pertinent role in the inflammatory response, including initiation of the acute-phase response and increasing metabolic risks as well as vascular plaque formation (36). Peripherally, IL-6 activates the production of inflammatory cytokines by initiating intracellular signaling with TLR (37). IL-6 is linked to substantial morbidity risks through arterial plaque formation, which can lead to cardiovascular disease and risks, high BMI, chronic pain, and an elevated risk for diabetes (38–42). Therefore, our findings suggest that improved sleep quality plays an important role in decreasing inflammation through gene-expression networks, which may also have a health-promoting role over time.

IL-6 also crosses the blood–brain barrier and contributes to central inflammation, and higher activity can contribute to mood alterations including symptoms of depression and anxiety (36). Here, we report depression symptoms decrease following improved sleep, and there is a trend for decreases in PTSD symptoms. Prior studies link inflammation to the onset of depressive symptoms, suggesting a complex bi-directional relationship that contributes to mental well-being (43, 44). Higher concentrations of IL-6 as well as increased gene-activity are established bio-markers for depression (45), yet, less is known about the inflammatory mechanisms that result from the interaction of insomnia and depression, and the resolution of symptoms. Previously, we reported that depression and insomnia in military personnel are associated with higher concentrations of CRP (23), and that improved sleep reduces inflammation (24). Here, we extend these findings by linking gene expression to sleep improvement and reduction in depression symptoms. In addition, we report a significant reduction in the expression of the CCL4 gene, which was reduced by −3.39 fold in the improved sleep group. CCL4 is an initiator of the inflammatory processes, suggesting inflammatory pathways are reduced in activity following sleep restoration and reduction in depression symptoms. Greater CCL4 activity is linked to major depressive disorder in both adolescents and adults (46, 47); however, it is not clear if the activity of this gene or other inflammatory initiating genes change following sleep improvement and mood change. Therefore, our findings of reductions in CCL4 following sleep improvement are novel and provide some initial insights into complex relationships among genes that regulate inflammation, sleep, and mood.

The current study also found a significant increase in the expression of 104 genes, with most being related to the regulation of inflammatory activity (Table 5). Of these up-regulated genes, the highest fold change is in pyruvate dehydrogenase kinase, isozyme 4 (PDK4), with a 3.12-fold change in the improved sleep group. This gene is of interest as it is located in the matrix of the mitochondria. PDK4 reduces the conversion of pyruvate, which is produced from the oxidation of glucose and amino acids, to acetyl-CoA. It also contributes to the regulation of glucose metabolism and has ramifications for overall health and morbidity. PDK4 has not been previously linked to sleep disturbance or mood. Here, we report that this gene increases expression in the context of reduced expression of genes that influence inflammation and contribute significantly to metabolic risk. Exercise alters the activity of PDK4, which relates to reductions in IL-6 activity, suggesting that PDK4 is involved in metabolic processes that are important to maintaining health (48). PDK4 is also implicated in the maintenance of body weight during periods of fasting, as it is required to maximize glucose metabolism; however, when animals are exposed to high-caloric diets that are unrestricted, PDK4 increases in expression (49). Therefore, PDK4 has complex relations to comorbid conditions of type 2 diabetes, cardiovascular disease, and other conditions that are common in military personnel and impact their morbidity. For these reasons, additional studies are needed to determine how this gene relates to sleep and mood, as well as morbidity risk in future studies.

In this study, we report two main gene-networks that are implicated in sleep improvement. The first involves the key regulator CTNNβ1, a gene that relates to the activity of both IL-2 and TLR4, as well as ubiquitin genes (Figure 1). The CTNNβ1 is most often implicated in vulnerability to and recovery from cancer (50–52); however, the role of this gene in sleep quality has not yet been determined. This gene encodes for the β-catenin protein, which regulates cell development and adhesion between cells, in turn creating and preserving epithelial cell layers, which is related to inflammatory gene activity. Activation of the Wnt signaling pathway, which is a pertinent system for cell-to-cell communications and which becomes activated during inflammatory responses, is essential in maintaining immune function system balance between inflammatory and anti-inflammatory activities. The result is increased concentrations of the protein that is coded by CTNNβ1 gene, which is linked to both increases in symptoms of depression and anxiety in clinical samples (53). Of importance, we also report an increase in IL-2 gene expression, which is a primary anti-inflammatory cytokine and is important for regulating inflammatory cytokine activity including IL-1β, IL-6, and TNF. Therefore, an increase in the expression of the IL-2 gene in conjunction with other inflammatory regulators suggests that regulatory gene networks are altered following sleep restoration, and likely contribute to our previous finding of reductions in CRP following improved sleep (23). Thus, these gene-expression findings together suggest that there is an alteration in the activity of the acute-phase response, which is responsible for the initiation of CRP production. Although we did not find that CRP significantly reduced in activity, our finding of reductions in the activity of these precursor genes that are more easily reduced in activity suggests that changes in CRP gene-activity may require more time to observe. Although our findings provide some key insights into the role of inflammation following sleep change, much less is known about the long-term impact of sleep disturbance on morbidity risks in military personnel and veterans.

We also report that TLRs, and specifically TLR4 gene expression is increased in the improved sleep group, and is also a key gene-network regulator of inflammatory activities. TLR4 recognizes pathogen-associated molecular patterns that are expressed by infectious agents, and mediate the production of cytokines necessary for the development of effective immunity, thereby regulating inflammatory cytokine activities following a stimulus. TLR4 has been linked to repeated hypoxia events in a pre-clinical model, suggesting that acute sleep deprivation may increase TLR activity (54). Our findings of an increase in TLR4 following improved sleep in conjunction with reduced activities of inflammatory cytokines suggests that the activities of TLR4 are impacted, contributing to a restoration of inflammatory immune cell function. Since UBC is tightly related to TLR4 in our gene-network, it may be that the protein-interaction properties of UBC that results in lessened gene activity may contribute to this immune profile in gene expression. It may also be that different molecular regulation mechanisms, including DNA methylation or other gene-silencing processes, are occurring. Nonetheless, the presence of TLR4 in this sample suggests it aided in the reduction of inflammation, as well as interacting with other pathways within the improved sleep group.

Our findings also implicated the protein UBC pathway in gene expression following improved sleep (Figure 2). Protein UBC is required for the removal of oxidized or misfolded proteins that are a consequence of neuronal activity that occurs during waking hours; it also alters the location of the protein in the cell or prevents protein interactions. In pre-clinical models, substantial sleep deprivation results in an accumulation of these bi-products within neurons, and subsequent neuronal damage (55). Accumulation of over-oxidized proteins is linked to cognitive impairments in insomnia patients (56). Therefore, there is an important role for ubiquitin in sleep regulation, which has been recently described in pre-clinical models including *Drosophila*, linking a knockdown of the E3 ubiquitin ligase Cul3 to sleep disruption (57). Reductions in the activity of E3 ubiquitin ligase Cul3 in neurons resulted in the insomniac phenotype (55). In mice with a spontaneous deletion in the ubiquitin carboxyl-terminal hydrolase L1 gene, there was a lower number of orexin A-immunoreactive neurons in the lateral hypothalamus and greater disruption in circadian rhythm compared to wild-type mice (58). Thus, our findings, within the context of previous studies, suggest that UBC genes are key mediators of sleep-related gene-activity changes.

Our findings have clinical relevance, as previous studies report that insomnia tends to precede depression and PTSD and that treatment of sleep disturbances decreases disease severity (59). Second, because of the stigma associated with mental illness, military personnel are more likely to seek treatment for sleep disturbances than for PTSD (14). Treatment for depression and PTSD symptoms results in substantial financial cost that may be mitigated by providing sleep interventions. In support of this, a comprehensive study undertaken by the RAND corporation estimates that treatment for either major depression or PTSD costs between $5,000 and $15,000 for each military personnel treated over 1 year (60). Lastly, our finding that even short-term improvement in sleep has a significant impact on inflammatory gene-activity, as well as reductions in depression symptoms, suggests that early assessment and treatment for insomnia following deployment is warranted, especially in military personnel with mood declines.

While our study has strengths, including a validated sleep measure, evaluation of comorbid symptoms, and a longitudinal design, there are some notable limitations. These include a relatively small sample of military personnel recruited from the same treatment facility. In addition, our sample primarily consisted of male participants and therefore cannot be generalized to women. Although we used validated questionnaires to determine PTSD and depression symptoms, these instruments did not provide a definitive clinical diagnosis. Lastly, other variations such as prescribed medications used by patients prior to the study and dose of treatment, including the number of treatment sessions attended and other interventional methods could have further impact on gene expression; however, the aim of this study is to focus on symptom and gene-expression changes, not to validate a standard of care treatment. In the future, a larger sample size and better estimate of treatment adherence and fidelity among participants are needed to confirm these findings.

Our novel findings suggest improved sleep quality has a biological correlate as well as a correlation with psychological symptoms. Less is known regarding the long-term impact of sleep disturbance on morbidity risks in military personnel and veterans; however, our findings suggest that improving sleep quality reduces inflammatory risks and psychiatric symptom severity. Therefore, our findings provide fundamental insights related to the connections between sleep improvement and gene expression, including inflammatory gene-activity, which may be used to reduce morbidity risks in military personnel following deployment. Improving assessment and treatment of sleep disturbance in this at-risk group may provide a novel health promotion opportunity.

## Conflict of Interest Statement

The authors declare that the research was conducted in the absence of any commercial or financial relationships that could be construed as a potential conflict of interest.

## Supplementary Material

The Supplementary Material for this article can be found online at http://journal.frontiersin.org/article/10.3389/fpsyt.2015.00059/abstract

Figure S1 **This network shows genes related to cell-mediated immune responses, as reported by IPA**. Reprinted with permission from QIAGEN’s Ingenuity^®^ Pathway Analysis (IPA^®^) (http://www.ingenuity.com/).

Figure S2 **This network shows genes related to connective tissue disorders, as reported by IPA**. Reprinted with permission from QIAGEN’s Ingenuity^®^ Pathway Analysis (IPA^®^) (http://www.ingenuity.com/).

Figure S3 **This network shows genes related to cellular function and maintenance and cellular development, as reported by IPA**. Reprinted with permission from QIAGEN’s Ingenuity^®^ Pathway Analysis (IPA^®^) (http://www.ingenuity.com/).

Figure S4 **This network shows genes related to organismal injury and abnormalities, as reported by IPA**. Reprinted with permission from QIAGEN’s Ingenuity^®^ Pathway Analysis (IPA^®^) (http://www.ingenuity.com/).

## Abbreviations

BMI, body mass index; CBT, cognitive behavioral therapy; CCL4, chemokine (C-C motif) ligand 4; CRP, C-reactive protein; CTNNβ1, catenin (cadherin-associated protein), beta 1, 88 kDa; FDR, false discovery rate; GC, GeneChip; IL-1β, interleukin 1 beta; IL-6, interleukin 6; IL-8, interleukin 8; IL-13, interleukin 13; IPA, ingenuity pathway analysis; NFATC2, nuclear factor of activated T-cells; OSA, obstructive sleep apnea; PCL-M, posttraumatic stress disorder checklist-military version; PDK4, pyruvate dehydrogenase kinase, isozyme 4; PKS, polyketide synthase; PSQI, the Pittsburg sleep quality index; PTSD, posttraumatic stress disorder; QIDS, quick inventory of depressive symptomatology; REML, restricted maximum likelihood; RIN, RNA integrity numbers; RMA, robust multi-array average; TLR1, toll-like receptor 1; TLR4, toll-like receptor 4; TLR7, toll-like receptor 7; TLR8, toll-like receptor 8; TNF, tumor necrosis factor; UBC, ubiquitin.
