# Supplementary figures and images for: Improved Sleep in Military Personnel is Associated with Changes in the Expression of Inflammatory Genes and Improvement in Depression Symptoms
Source: Front Psychiatry. 2015 Apr 30;6:59. doi: 10.3389/fpsyt.2015.00059 (PMC4415307; doi:10.3389/fpsyt.2015.00059)

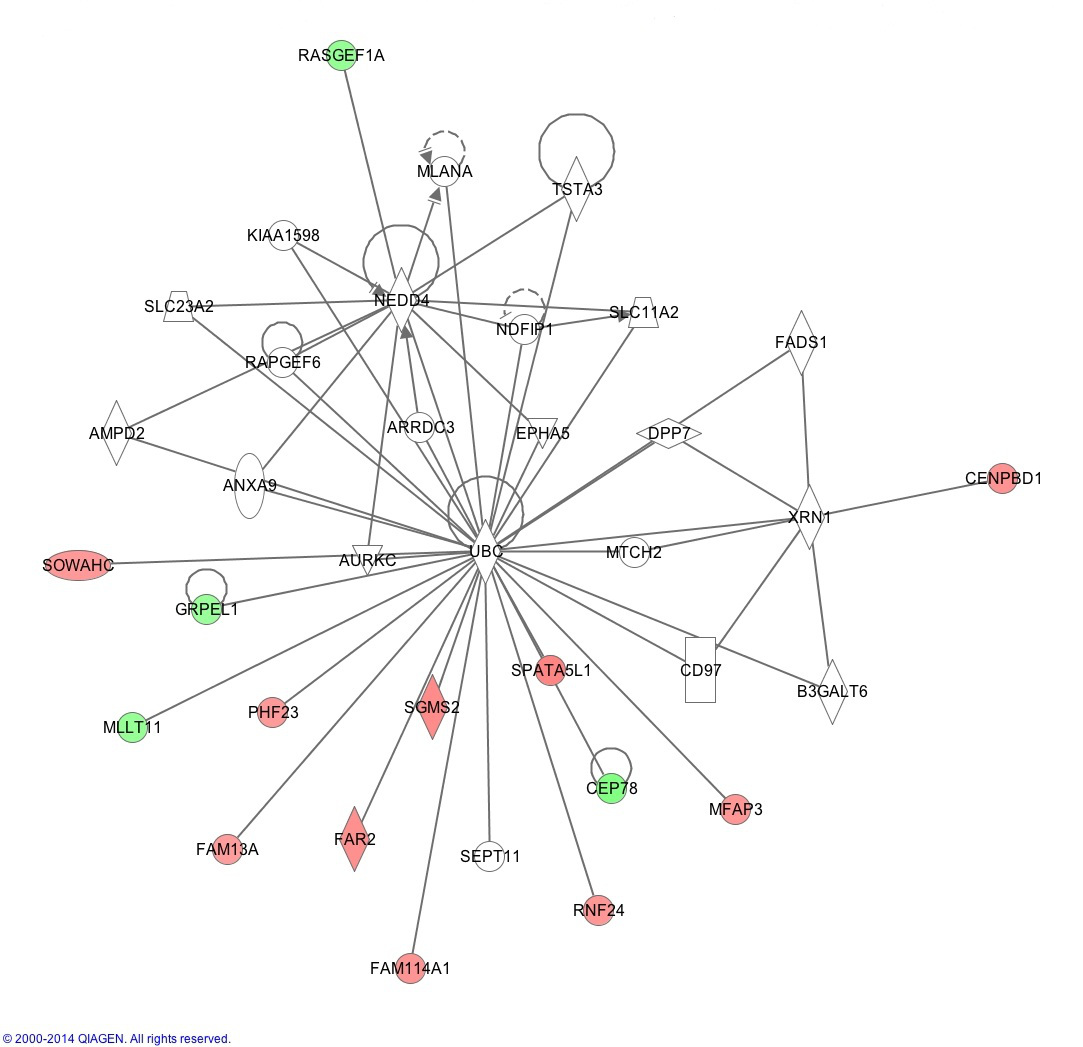

Supplement: Figure S1 — This network shows genes related to cell-mediated immune responses, as reported by IPA. Reprinted with permission from QIAGEN’s Ingenuity® Pathway Analysis (IPA®) (http://www.ingenuity.com/). [file Image_1.JPEG]

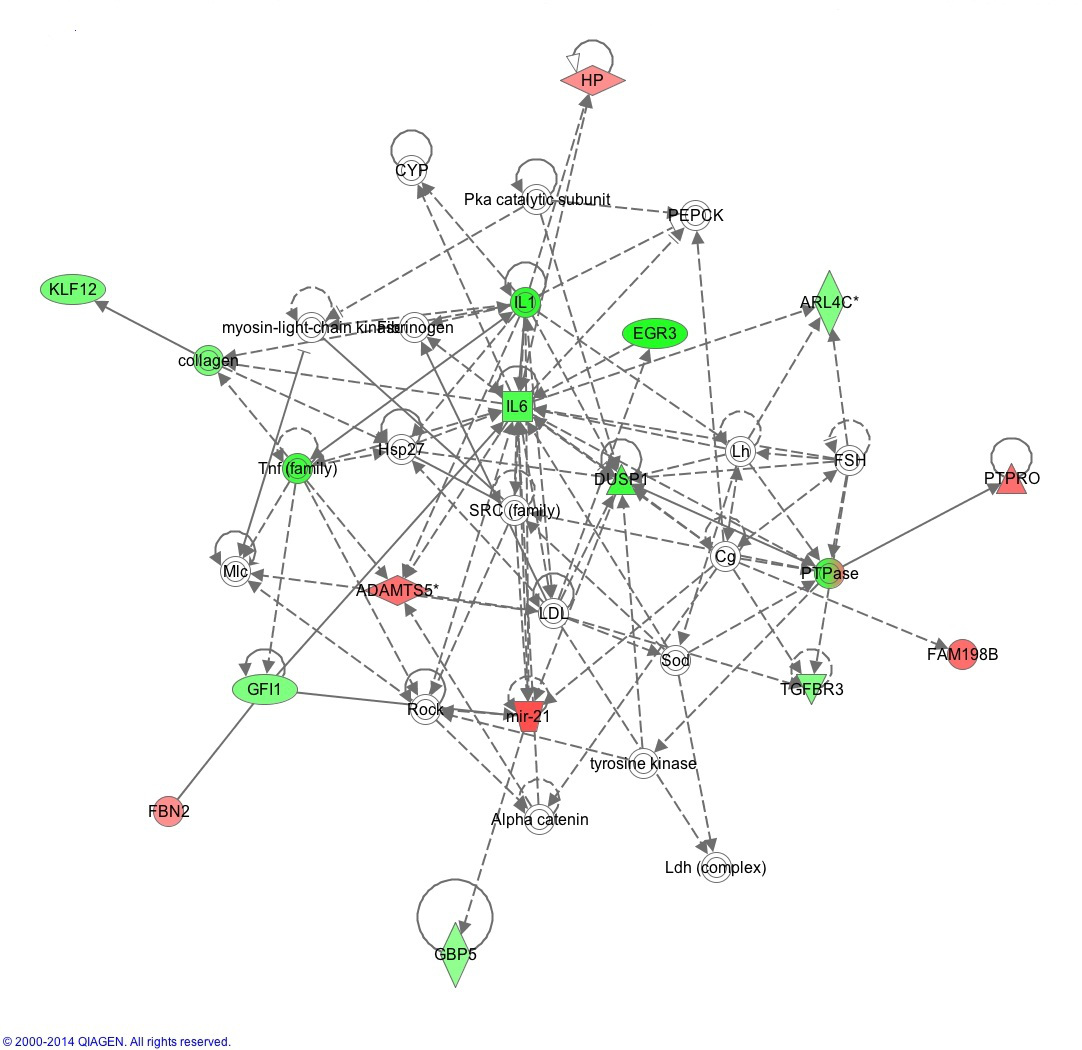

Supplement: Figure S2 — This network shows genes related to connective tissue disorders, as reported by IPA. Reprinted with permission from QIAGEN’s Ingenuity® Pathway Analysis (IPA®) (http://www.ingenuity.com/). [file Image_2.JPEG]

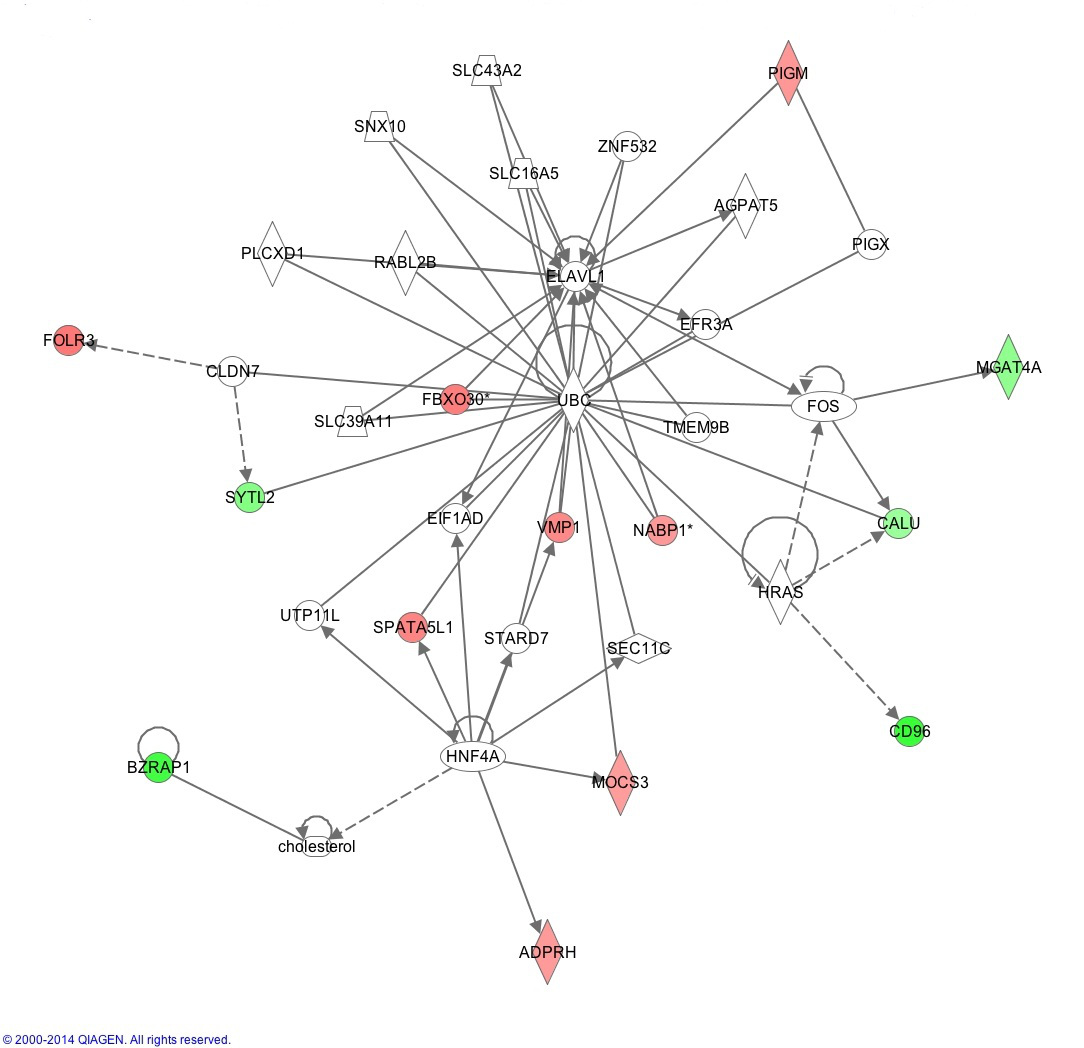

Supplement: Figure S3 — This network shows genes related to cellular function and maintenance and cellular development, as reported by IPA. Reprinted with permission from QIAGEN’s Ingenuity® Pathway Analysis (IPA®) (http://www.ingenuity.com/). [file Image_3.JPEG]

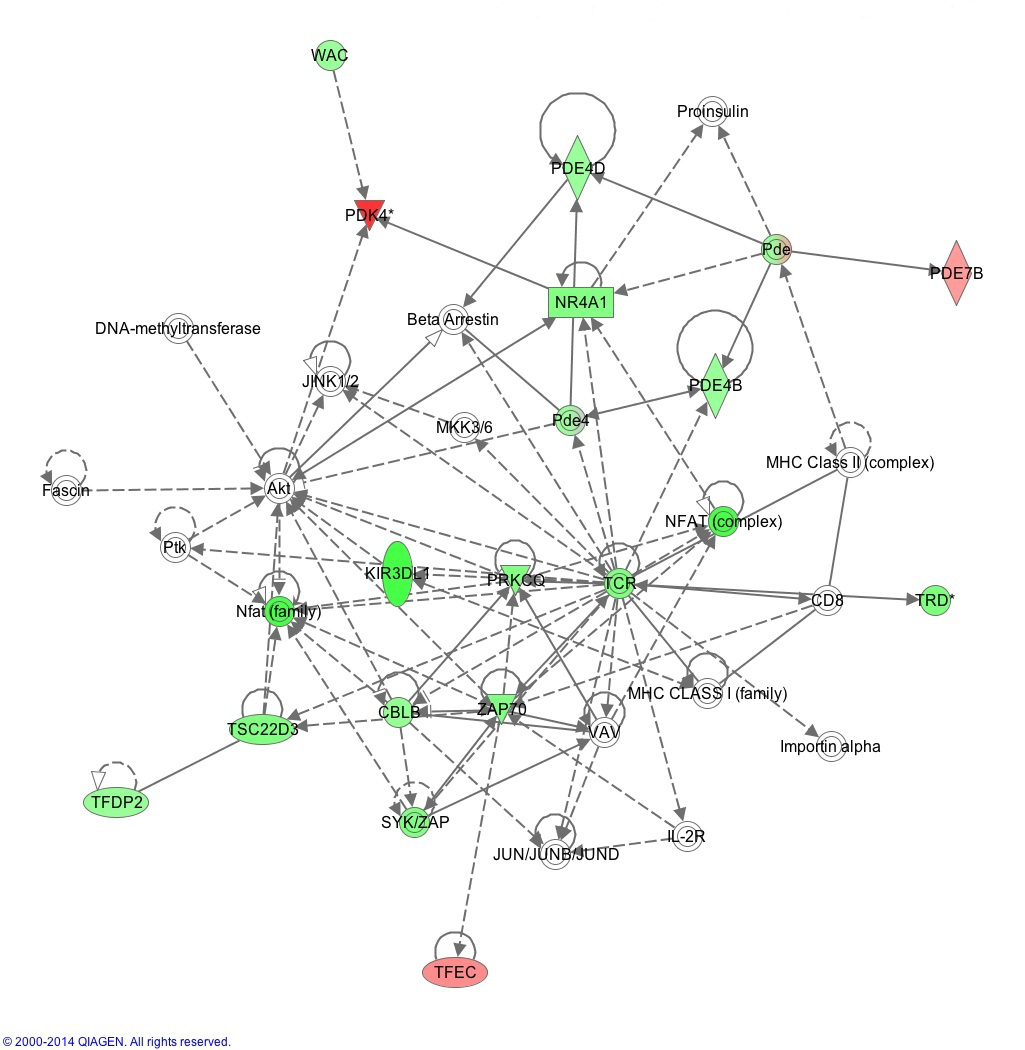

Supplement: Figure S4 — This network shows genes related to organismal injury and abnormalities, as reported by IPA. Reprinted with permission from QIAGEN’s Ingenuity® Pathway Analysis (IPA®) (http://www.ingenuity.com/). [file Image_4.JPEG]
